# A permeability assay for mouse intestinal organoids

**DOI:** 10.1016/j.xpro.2022.101365

**Published:** 2022-05-01

**Authors:** Stijn Aaron den Daas, Ugo Soffientini, Shilpa Chokshi, Gautam Mehta

**Affiliations:** 1 Roger Williams Institute of Hepatology, Foundation for Liver Research, London SE5 9NT, UK; 2 School of Immunology and Microbial Diseases, Faculty of Life Sciences and Medicine, King’s College London, London WC2R 2LS, UK; 3 Institute for Liver and Digestive Health, Royal Free Campus, University College London, London NW3 2PF, UK

**Keywords:** Health Sciences, Model Organisms, Organoids

## Abstract

Here, we describe an assay for intestinal permeability in mouse intestinal organoids, although this may also be adapted for other species. Propidium iodide (PI) does not penetrate intact biological membranes and thus cannot enter the lumen of intact organoids. Passage of PI within the lumen can be induced by tight junction disruption or epithelial cell death. This technique measures PI-stained extruded dead cells within the organoid lumen to analyze the effect of insults, toxins, or treatments on intestinal organoid permeability.

## Highlights

• This protocol allows measurement of intestinal permeability in organoids
• The permeability assay is scalable and reproducible
• Allows assessment of drugs targeting intestinal epithelial permeability
• Knockout mouse strains may also be used to dissect pathobiology of permeability

## Graphical abstract

**Figure:**
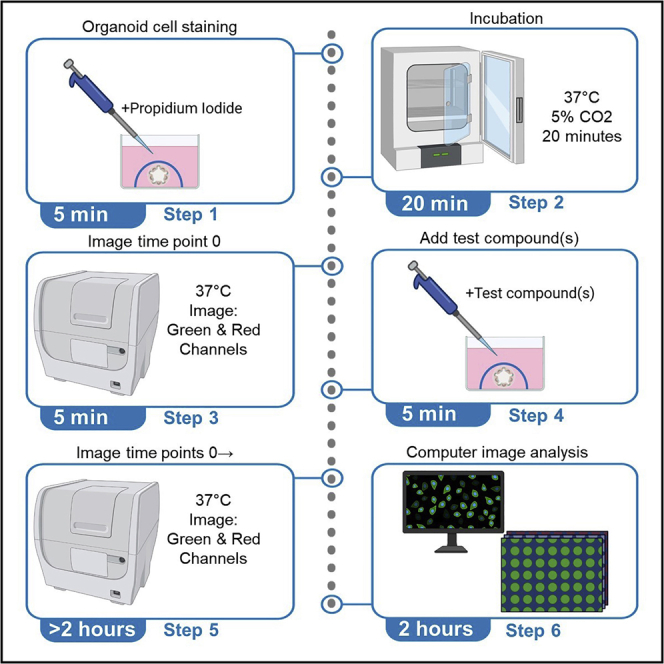

## Before you begin

The protocol below describes the specific steps to measure permeability of intestinal organoids derived from mouse intestines, however this protocol could also be used to measure permeability intestinal organoids derived from other species.

Intestinal organoid derival and culture needs to be set up prior to initiating this protocol. This permeability assay rapidly measures the permeability of live organoids in a matter of hours. Therefore, the intestinal organoids need to be plated and grown prior to the assay according to the researchers’ requirements, i.e., at least one day to analyze the effect of a substance on permeability of growing intestinal organoids, or 3–6 days on fully grown intestinal organoids. Plated intestinal organoids should be at a density to allow individual organoid imaging without overlapping organoids to simplify imaging analysis (∼50–100 organoids per dome). We plate a single 30 μL Matrigel dome with intestinal organoids in a 24-wells plate with 500 μL medium to measure permeability, but this can be scaled up or down depending on experimental requirements. Additional wells with organoids in the experiment can be used as a negative control, positive permeabilized control (e.g., ethylenediaminetetraacetic acid, EDTA), and positive cell death control (e.g., Triton X-100).

### Intestinal organoid derival and culture

**Timing: 2–3 weeks**

1. Derive and culture intestinal organoids from mouse intestine samples (Sato et al., 2011).

***Note:*** A few passages before conducting this permeability assay are advised to reduce dead intestinal epithelial cell debris from the culture and to verify long term viability of the organoids.

2. Freshly plate one 30 μL Matrigel dome with 50–100 organoids per well of a 24-well plate for each treatment (plus 3 wells for controls) with 500 μL intestinal organoid growth medium, and culture 3–6 days to grow out the intestinal organoids into budding-spherical intestinal organoids. Replenish organoid growth medium every 2 days.

## Key resources table

**Table:** 

REAGENT or RESOURCE	SOURCE	IDENTIFIER
**Chemicals, peptides, and recombinant proteins**		

HOECHST 33342	Thermo Fisher Scientific	Cat#H1399
Propidium Iodide Solution	Merck	Cat#P4864
Corning Matrigel Growth Factor Reduced Basement Membrane Matrix, phenol red-free	VWR	Cat#734-1101
Gibco DMEM/F12, HEPES	Thermo Fisher Scientific	Cat#11330032
N-2 Supplement (100×)	Thermo Fisher Scientific	Cat#17502048
B-27 Supplement (50×)	Thermo Fisher Scientific	Cat#17504044
N-Acetyl-L-cysteine	Merck	Cat#A9165
Recombinant murine EGF	PeproTech	Cat#315-09
Recombinant murine Noggin	PeproTech	Cat#250-38
Recombinant human R-Spondin-1	PeproTech	Cat#120-38
Penicillin Streptomycin	Thermo Fisher Scientific	Cat#15140122
EDTA	Merck	Cat#ED2SS
Triton X-100	Merck	Cat#X100
Penicillin-Streptomycin	Merck	Cat#P4333

**Experimental models: Organisms/strains**		

Mouse (*Mus musculus)*: C57BL/6J wild type,∼15 weeks old, female	The Jackson Laboratory	JAX: 000664

**Critical commercial assays**		

LDH-Glo Cytotoxicity assay	Promega	Cat#J2380

**Software and algorithms**		

Fiji (or ImageJ)	ImageJ	https://imagej.net/software/fiji/

**Other**		

Cytation 5	Agilent (BioTek)	https://www.biotek.com/products/imaging-microscopy-cell-imaging-multi-mode-readers/cytation-5-cell-imaging-multi-mode-reader/overview/

## Materials and equipment

**Table:** Intestinal organoid growth medium

Reagent	Final concentration	Amount
Gibco DMEM/F12, HEPES (2/8°C)	n/a	480 mL
N-2 Supplement (100×) (−5/−20°C)	1×	5 mL
B27 Supplement (50×) (−5/−20°C)	1×	10 mL
N-Acetyl-L-cysteine (2°C–8°C)	1 mM	81.5 mg
Recombinant murine EGF (−20°C)	50 ng/mL	25 μg
Recombinant murine Noggin (−20°C)	100 ng/mL	50 μg
Recombinant human R-Spondin-1 (−20°C)	1 μg/mL	500 μg
Penicillin (10.000 U/mL), Streptomycin (10.000 μg/mL) (−5/−20°C)	100 U/mL, 100 μg/mL	5 mL
**Total**	**n/a**	**500 mL**

Intestinal organoid growth medium needs to be stored between 2°C and 8°C. Some components have a short shelf life at 4°C, therefore stock solutions can be aliquoted in order to produce less fresh medium every week.

**CRITICAL:** N-Acetyl-L-cysteine can cause serious eye irritation. Personal protective equipment needs to be worn according to local health and safety requirements.

***Alternatives:*** Conditioned medium containing EGF, Noggin, and/or R-Spondin-1 producing cells can be used in the medium instead of Recombinant murine EGF/Recombinant murine Noggin/Recombinant human R-Spondin-1 (Cattaneo et al., 2020). A comparison between the intestinal organoid growth media is than required to verify organoid viability.

## Step-by-step method details

### Organoid cell staining

**Timing: 30 min**

This step is to stain the dead intestinal organoid cells; propidium iodide (PI) is cell impermeable and therefore only stains dead cells. Dead cells within the intestinal organoid lumen are shielded from PI by the intestinal monolayer, and thus will not be stained by PI without an increase in organoid permeability (Bode et al., 2019).

1. Mix PI solution (5 mg/mL stock) in pre-warmed intestinal organoid growth medium with a final concentration of 5 μg/mL PI.
2. Replace existing media from the well with the media with PI, and incubate at 37°C for 20 min.

***Note:*** PI (and HOECHST) are added to intestinal organoid growth medium rather than PBS to maintain organoid health.

***Optional:*** HOECHST can be used to stain alive and dead cells, since HOECHST is cell permeable. This is, however, not required to measure permeability, but can give an image of the entire intestinal organoid. HOECHST staining is toxic for intestinal organoids after prolonged exposure and therefore is not recommended in this protocol when measuring permeability for more than 12 h. When additionally staining with HOECHST, add HOECHST (5 mg/mL stock) to the pre-warmed intestinal organoid growth media with a final concentration of 5 μg/mL during step 1.

### Organoid imaging and permeability analysis

**Timing: 1 day**

**Figure 1:**
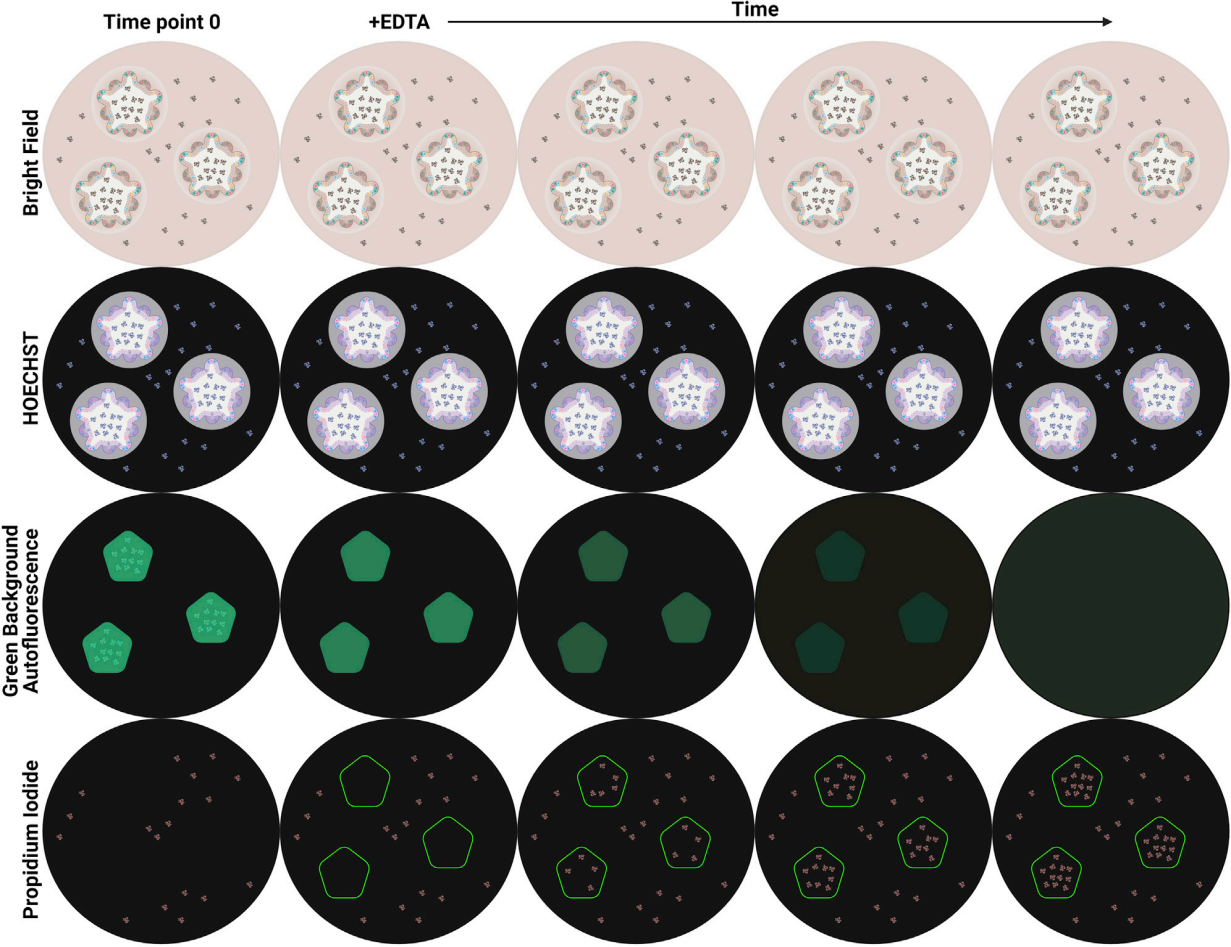
Illustrations of expected EDTA treated intestinal organoids images Illustrations of bright field, HOECHST, green background autofluorescence (AF), and propidium iodide (PI) images of intestinal organoids treated with EDTA over time. In the PI images, the luminal areas of intestinal organoids are highlighted, which are made by selecting the luminal region in the green background AF. Analyzing PI stain over time in these luminal regions are indicative of organoid permeability.

This step is to image the intestinal organoids in green (469–525 nm) and red (531–593 nm) channels to measure green background autofluorescence (AF) and PI respectively. By measuring PI stain within the organoid lumen at the same spot pre and post treatment, a toxin/compound’s effect on intestinal organoid permeability can be analyzed. The lumen of intestinal organoids can be defined by the green background AF of dead cells and other debris inside the organoid lumen (Bardenbacher et al., 2020). The green background AF inside the lumen decreases with permeability, however, analyzing green background AF inside the lumen pre- and post- treatment was found to be less precise, and also more susceptible to bleaching and other processes (unrelated to permeability) that can interfere with this measurement compared to analyzing PI intensity inside the organoid lumen (Figure 1).

3. Place the intestinal organoid plate into the Cytation 5 and image a Z stack of the intestinal organoids at 4× magnification in green 469–525 nm (luminal extruded dead cell debris) and red 531–593 nm (PI) channels for time point 0.

***Note:*** Imaging the organoids in focus can be difficult because of the 3D structure of organoids, therefore we recommend imaging a Z stack every time which can be stacked in Fiji and Z projected later to acquire a clear in-focus image of all the organoids.

***Optional:*** Bright field images can be taken during step 3.

***Optional:*** Imaging the blue (377–447 nm) channel is required during step 3 when also staining with HOECHST**.**

***Alternatives:*** An alternative automated fluorescent microscope, instead of the Cytation 5, that can image repeatedly on the same x-y-z axis can also be used (e.g., ImageXpress Nano Automated Imaging System), but requires optimization for this protocol.

4. Add the test toxin/compound(s), control (negative), EDTA (final concentration 2 mM, positive permeable control), and Triton X-100 (final concentration 0.5%, positive cell death control) to each single well with intestinal organoids.
5. Collect a sample of medium (2 μL) for a lactate dehydrogenase (LDH) assay before taking each image.
6. Place the intestinal organoid plate back into the Cytation 5 and image the intestinal organoids at the same spots in the same channels after treatment.

***Note:*** For the positive EDTA control, and positive Triton X-100 control imaging 150 min is sufficient to analyze permeability, however, longer measurement may be required before organoids become permeable, depending on the toxin/compound’s mode of action.

7. Determine degree of cell death by measuring LDH release.

***Note:*** The LDH assay, which measures LDH in the supernatant and is a marker for plasma membrane damage, allows this protocol to differentiate between increased permeability by cell death and increased permeability by decrease in intercellular junctions.

***Alternatives:*** A different cell death assay to LDH release can be used to analyze cell death in organoids, but needs optimization to work with 3D organoids.

**Pause point:** After the LDH release measurement (step 7) is a good pause point, since the following steps are analysis only and can therefore be performed at a later time once the images are acquired.

8. Open the green channel image of time point 0 as a stack in Fiji (see Figure 2).
  a. “Z Project” the stack to get all organoids of the stack in focus (Image, Stacks, Z Project…, Max Intensity).
  b. Set the “Threshold” (Image, Adjust, Threshold) so that only green background AF of dead cell debris in the organoid lumen is selected.
  c. Select this region of the binary image by “Create Selection” (Edit, Selection, Create Selection).

**Figure 2:**
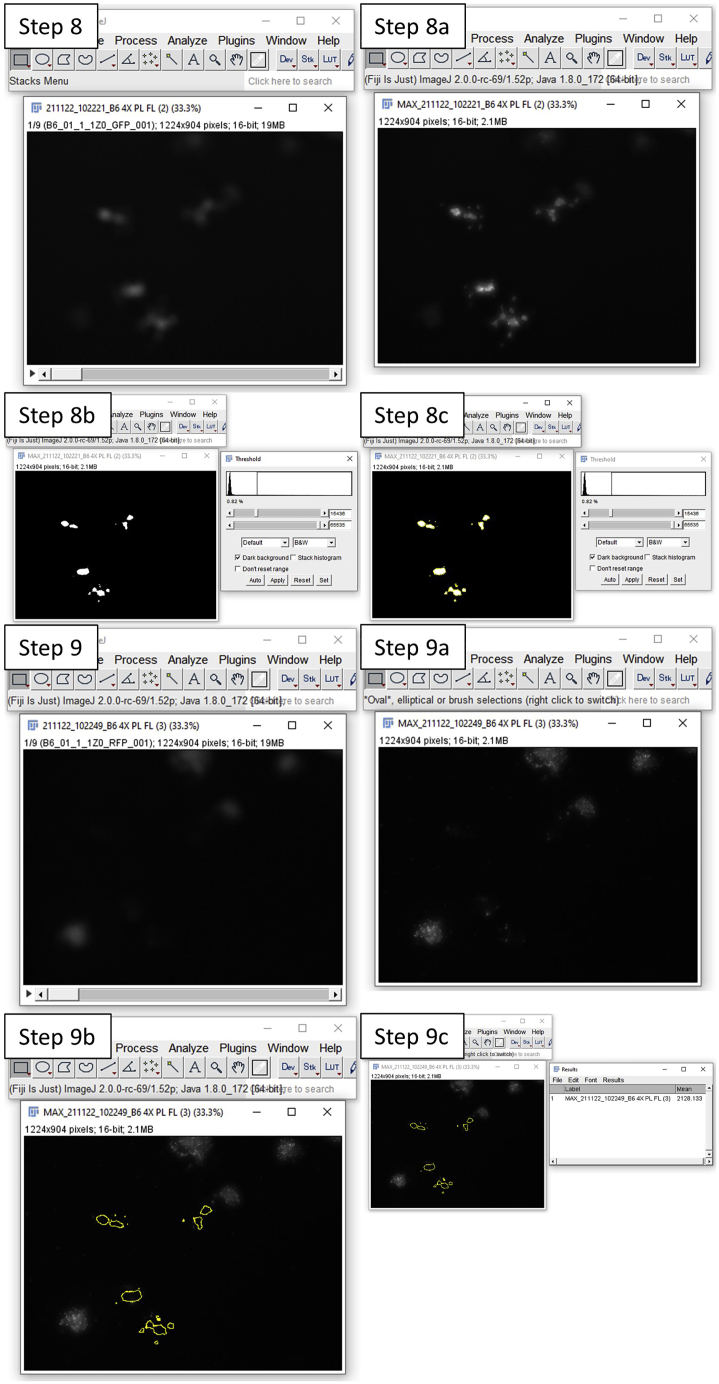
Screenshots from Fiji image capture
9. Open the red channel image of time point 0 as a stack in Fiji.
  a. “Z Project” the stack to get all organoids of the stack in focus (Image, Stacks, Z Project…, Max Intensity).
  b. Paste the previously selected region into this image (Control + Shift +E).
  c. Measure the mean intensity of PI in this region by “Measure” (Analyze, Measure).

***Optional:*** Permeability can be measured at multiple time points by repeating steps 5–9 for multiple time points.

10. Open the other time points of the red channel and repeat step 9.
11. Compare the mean intensity of PI stain of dead cells inside the selected organoid lumen of treated organoids to control (untreated) organoids.

***Note:*** By measuring mean PI intensity inside the selected organoid lumens at time point 0 the size and number of organoids are normalized and are equalized at time point 0. By comparing treated to control luminal mean PI intensity after treatment time any change in background fluorescence is accounted for. When analyzing data after a pre-selected time point a parametric/non-parametric test can be used, e.g., t-test or ANOVA.

***Alternatives:*** The ratio of luminal mean PI intensity after treatment to time point 0 can also be used to compare treated to control organoids for permeability.

## Expected outcomes

**Figure 3:**
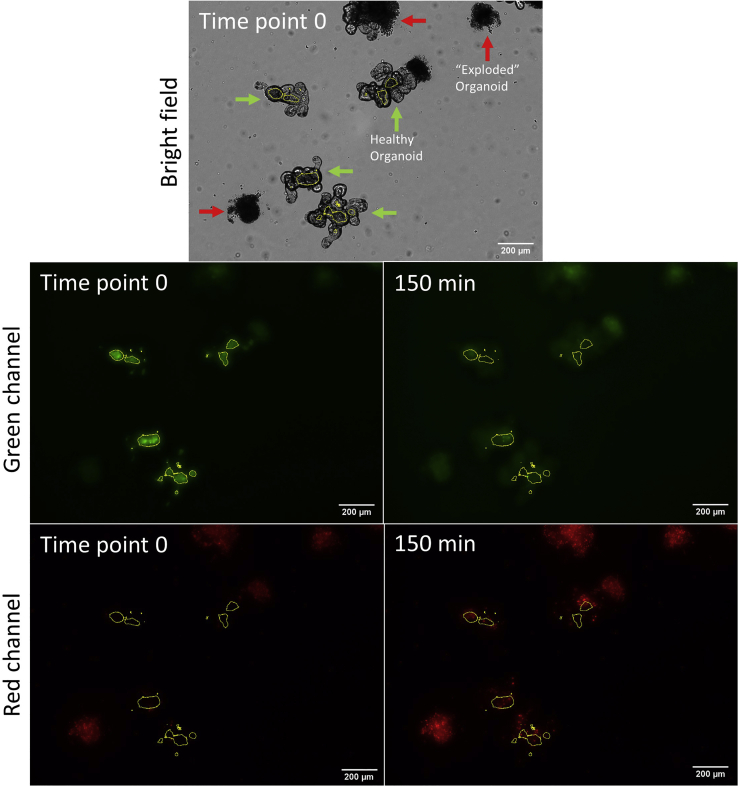
Intestinal organoids treated with EDTA Mouse intestinal organoids were treated with 2 mM EDTA and imaged at time point 0 and every 30 min for 150 min. A bright field image at time point 0, green channel images of time point 0 and after 150 min, and red channel images from time point 0 and after 150 min are shown with luminal region selected from the green channel from time point 0.

**Figure 4:**
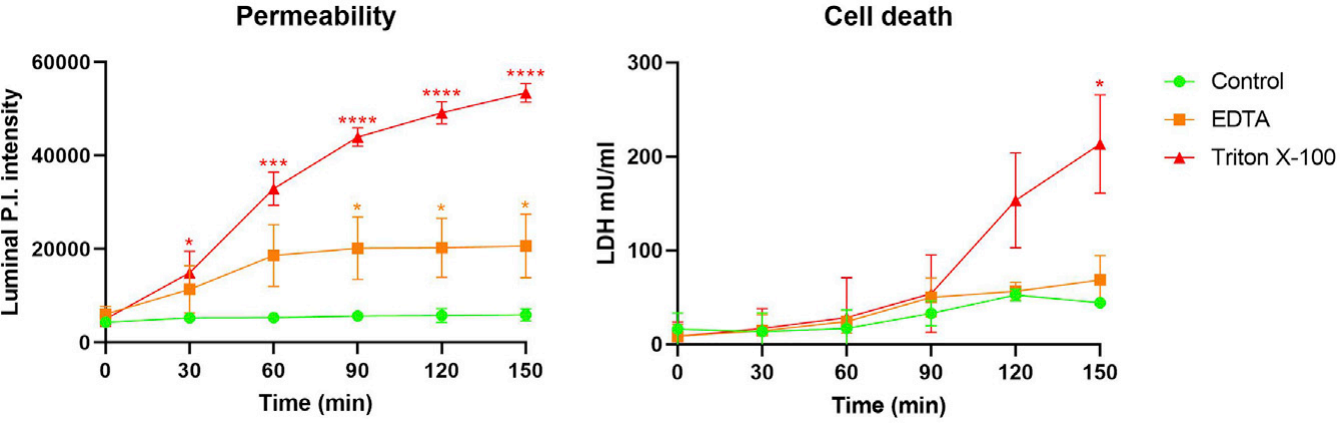
Results of luminal propidium iodide and LDH measurements of intestinal organoids Luminal propidium iodide stain and LDH release of intestinal organoids treated with 2 mM EDTA, and 0.5% Triton X-100 were measured over time using this intestinal organoid permeability assay and compared to control. Statistics calculated using two-way ANOVA with post-hoc Tukey test, ∗p<0.05, ∗∗p<0.01,∗∗∗p<0.005, ∗∗∗∗p<0.0001, n=4, data are represented as mean ± SD.

Using this protocol, the effect of a toxin or compound on intestinal organoid permeability can be measured over time. Untreated intestinal organoids are not expected to show an increase in organoid permeability and will show a horizontal line of luminal PI accumulation over time (Figures 3 and 4).

*In vitro* experiments have shown that EDTA disrupts tight junctions and induces paracellular pores that facilitate transport exclusively through the paracellular pathway in cell monolayers (Wang et al., 2016). In intestinal epithelial adenocarcinoma cells (Caco-2), the mechanism of EDTA disruption of tight junctions has been shown to be through calcium chelation, leading to an increase in paracellular transport (Tomita et al., 1996). Consequently, EDTA has been used to loosen crypt cells from isolated intestines for organoid derival, and also to increase organoid permeability (Sato et al., 2011; Co et al., 2019). In this protocol, EDTA-treated intestinal organoids are expected to show an increase in organoid permeability in the absence of cell death. Therefore, the mean fluorescence intensity of PI stain within the organoid lumen of EDTA-treated intestinal organoids is expected to increase compared to control, while LDH release remains similar to control levels.

Finally, Triton X-100-treated intestinal organoids are also expected to show an increase in organoid permeability, however unlike EDTA-treated they will also show increased cell death. Triton X-100 induces a loss of inter- and intra-cellular integrity through detergent action, leading to increased cell permeability and cell death. PI thereby able to stain all organoid cells in Triton X-100-treated organoids.

## Quantification and statistical analysis

In this assay, the intestinal organoid lumen is defined by the green background AF of dead cells and debris within the lumen. This automatically excludes dead and/or damaged organoids which lack an intact lumen, since they do not show green background AF. Intestinal organoids that are newly-plated may lack the green background AF of dead cells and debris within the lumen since insufficient dead cells may have been extruded into the lumen; these organoids are thereby excluded from this assay. Setting the green fluorescence threshold of the image at time point 0 determines the organoid lumen area. This area is then overlaid onto the PI images to measure the intensity of PI-stained cells within the lumen. The data acquired from treated organoids should then be compared to control organoids to analyze the toxin/compound’s effect on intestinal organoid permeability.

## Limitations

Healthy and proliferating intestinal organoids are essential for this assay; consult the paper by Sato et al. regarding derivation and culture of intestinal organoids for issues with this (Sato et al., 2011). This assay requires green background AF of dead cells and debris inside the organoid lumen, which excludes analyzing permeability on other types of organoids that do not extrude dead cells into the organoid lumen. For example, intestinal organoids with the polarity reversed, as demonstrated by Co et al., cannot be analyzed with this assay (Co et al., 2019).

Compounds with a fluorophore that falls within the same emission wavelength as PI cannot be administered as this will interfere with the measurement of dead cells inside the organoid lumen. However, compounds with different fluorophores can be administrated to intestinal organoids to determine their effect on organoid permeability.

To measure PI-stained cells inside the organoid lumen, it is essential to image on the same spot as time point 0, as the lumen areas are defined at that time point. We utilize the Cytation 5, which can image at precise spots in a repeated manner over time; other automated fluorescence microscopes can be used as well, provided that the image location can be repeated.

This protocol determines the lumen by green background AF at time point 0 and measures PI intensity within that region pre and post treatment. Since the lumen will grow as the organoid is growing and expending, over time the lumen will be different from time point 0. Therefore, this protocol is not suitable to measure the effects of toxins or compounds on organoid permeability beyond 24 h of treatment.

## Troubleshooting

One of the challenges of this protocol is that the mode of action of each toxin/compound is different and any change in intestinal organoid permeability may take an unknown length of time. Therefore, an optimization experiment can be conducted with multiple images taken over several hours to pinpoint the duration of the toxin’s/compound’s effect. Subsequently, a duration can be chosen that is appropriate to analyze the effect of that particular toxin/compound on organoid permeability.

### Problem 1

No fluorescence signal can be seen in the images during step 3.

### Potential solution

If no fluorescence signal can be seen when imaging the stained intestinal organoids, increase the exposure. If no signal can be seen after increasing the exposure than check if the correct channel is set to image green background AF and/or PI (/HOECHST). PI (and HOECHST) has an excitation and emission curve that can be inspected beforehand to examine which fluorescence channel is best suited. For the green background AF, we have found the signal to be strongest in the GFP channel, but this can differ depending on the emission filter. PI (and HOECHST) is a common cell dye; if no fluorescence can be found at the correct channels, make sure the solutions are not expired or photobleached. Fluorophores can be photobleached if exposed to light for too long and then new dyes are required. If HOECHST and PI stain are not the problem and no green background AF can be seen then inspect the organoids health, since only intact intestinal organoids will produce green background AF. Examining green background AF of organoids at multiple time points after plating can help with determining the optimal growth duration before conducting the experiment.

### Problem 2

Intestinal organoids will not grow during intestinal organoid derival and culture (before you begin).

### Potential solution

Because intestinal organoids are derived from intestinal crypt stem cells, they lack the full intestinal microenvironment when cultured *in vitro*. Therefore, they require specific supplements to maintain their stemness and proliferation, as well as a gel matrix to support the 3D structure of the organoid. Consult the paper by Sato et al. about the specific supplements and concentrations for the different intestinal organoids (Sato et al., 2011). However, when using conditioned medium of EGF, Noggin, and/or R-Spondin-1 producing cells, compare the viability of intestinal organoid growth medium made with the conditioned medium with that of intestinal growth medium made with Recombinant EGF, Noggin, and R-Spondin-1. If the problem is caused by conditioned medium containing EGF, Noggin, and/or R-Spondin-1, try adjusting the ratio of conditioned medium to improve viability.

### Problem 3

Intestinal organoids do not show an increase in permeability at step 11.

### Potential solution

Not all toxins/compounds will increase intestinal organoid permeability. We advise the usage of EDTA as a positive control alongside the toxin/compound(s) to verify the result of the assay. We treat intestinal organoids with 2 mM EDTA, which show increased permeability within 2 h.

### Problem 4

Intestinal organoids die during the intestinal organoid permeability assay, between steps 3 and 7.

### Potential solution

Some toxins/compounds will be toxic to the intestinal organoid cells. We therefore measure LDH release into the supernatant (as a measure of lytic cell death) at several time points to differentiate between loss of inter-cell junctions and increased cell death in the permeability signal. HOECHST stain can also be toxic to organoids, therefore if analyzing permeability for a prolonged period eliminate HOECHST stain from the steps as this is not essential for determining organoid permeability.

### Problem 5

Time point 0 and after-treatment images do not line up, at steps 8–10.

### Potential solution

The use of an automated fluorescence microscope is needed to image the intestinal organoids in the same x-y-z axis pre and post treatment. However, if a drift in the x-y-z axis occurs or a manual fluorescence microscope is used and the pre and post treatment images do not line up, the selected green background AF region after pasting on the PI image can be adjusted by click and moving the region. This is a manual adjustment and is therefore error-prone, thus should be avoided if possible.

## Resource availability

### Lead contact

Further information and requests for resources and reagents should be directed to and will be fulfilled by the lead contact, Gautam Mehta (g.mehta@researchinliver.org.uk).

### Materials availability

This study did not generate new unique reagents.

## Data Availability

All data reported in this paper will be shared by the lead contact upon request. This paper does not report original code. Any additional information required to re-analyze the data reported in this paper is available from the lead contact upon request.
